# How, who, and when: preferences for delivery of genome sequencing results among women diagnosed with breast cancer at a young age

**DOI:** 10.1002/mgg3.254

**Published:** 2016-10-24

**Authors:** Kimberly A. Kaphingst, Jennifer Ivanovich, Ashley Elrick, Rebecca Dresser, Cindy Matsen, Melody S. Goodman

**Affiliations:** ^1^ Department of CommunicationUniversity of UtahSalt Lake CityUtah; ^2^ Huntsman Cancer InstituteUniversity of UtahSalt Lake CityUtah; ^3^ Division of Public Health SciencesWashington University School of MedicineSt. LouisMissouri; ^4^ Washington University School of LawSt. LouisMissouri; ^5^ Department of SurgeryUniversity of UtahSalt Lake CityUtah

**Keywords:** Breast cancer, genome sequencing, patient preferences, return of results, secondary findings.

## Abstract

**Background:**

The increasing use of genome sequencing with patients raises a critical communication challenge: return of secondary findings. While the issue of what sequencing results should be returned to patients has been examined, much less attention has been paid to developing strategies to return these results in ways that meet patients' needs and preferences. To address this, we investigated delivery preferences (i.e., who, how, when) for individual genome sequencing results among women diagnosed with breast cancer at age 40 or younger.

**Methods:**

We conducted 60 semistructured, in‐person individual interviews to examine preferences for the return of different types of genome sequencing results and the reasons underlying these preferences. Two coders independently coded interview transcripts; analysis was conducted using NVivo 10.

**Results:**

The major findings from the study were that: (1) many participants wanted sequencing results as soon as possible, even at the time of breast cancer diagnosis; (2) participants wanted an opportunity for an in‐person discussion of results; and (3) they put less emphasis on the type of person delivering results than on the knowledge and communicative skills of that person. Participants also emphasized the importance of a results return process tailored to a patient's individual circumstances and one that she has a voice in determining.

**Conclusions:**

A critical goal for future transdisciplinary research including clinicians, patients, and communication researchers may be to develop decision‐making processes to help patients make decisions about how they would like various sequencing results returned.

## Abstract 2

While the issue of what genome sequencing results should be returned to patients has been examined, much less attention has been paid to developing strategies to return these results in ways that meet patients' needs and preferences. To address this, we investigated delivery preferences (i.e., who, how, when) for individual genome sequencing results among women diagnosed with breast cancer at age 40 or younger. The major findings from the study were that: (1) many participants wanted sequencing results as soon as possible, even at the time of breast cancer diagnosis; (2) participants wanted an opportunity for an in‐person discussion of results; and (3) they put less emphasis on the type of person delivering results than on the knowledge and communicative skills of that person.

## Background

Whole exome and genome sequencing are rapidly being introduced into clinical settings (Guttmacher et al. 2010; Biesecker and Green 2014), and this expansion is expected to continue with advances in genomic technologies (Biesecker and Green 2014). Routine use of whole genome sequencing may soon become a reality in the clinic (Pasche and Absher 2011). Individual genomic information generated by sequencing has the potential to alter clinical care dramatically (Mardis 2008; Biesecker and Green 2014). The growing use of sequencing with patients raises a critical communication challenge: the return of secondary findings (Roberts et al. 2010; Kaphingst et al. 2016), that is, results not related to the indication for ordering sequencing but that may be of value or utility (Green et al. 2013). The issue of what secondary findings should be returned to patients has been actively debated, with an emerging consensus among experts that researchers and clinicians should offer to disclose analytically valid findings that are clinically actionable (Fabsitz et al. 2010; Fullerton et al. 2012; Green et al. 2013; Jarvik et al. 2014). In 2013, the American College of Medical Genetics and Genomics (ACMG) recommended that when clinical sequencing is performed, a minimum list of 56 genes should be evaluated and results returned to the ordering clinician (Green et al. 2013). However, much less attention has been paid to developing strategies to return secondary findings in ways that meet patients' needs and preferences.

Patient preferences could relate to different aspects of the delivery of genome sequencing findings, including the format for returning findings, the person who returns them, and the timing of the disclosure. Existing studies on delivery preferences for communication of different types of genetic and genomic test results have yielded mixed results. For delivery format, some studies have indicated a preference for written results (Shalowitz and Miller 2008; Streicher et al. 2011), whereas other studies have shown preferences for in‐person delivery with a written record (O'Daniel and Haga epub before press) or telephone or in‐person delivery (Fernandez et al. 2009; Christensen et al. 2011).

Studies that specifically address the return of sequencing results have also produced similarly mixed findings, with preferences expressed for in‐person, Internet‐based, mail, or phone delivery or for a combination of these methods (Yu et al. 2013, 2014; Bui et al. 2014; Levenseller et al. 2014; Wright et al. 2014). With respect to information source, prior research on return of genome sequencing findings has suggested preferences for various types of clinicians. For example, among focus group participants from the general public, doctors or physicians were more commonly identified, followed by genetic counselors, and then psychiatrists or therapists (Yu et al. 2014). Few studies have examined when patients would like to have their findings from genome sequencing returned, although concerns have been raised that patients may not be emotionally prepared to learn about secondary findings at an initial disclosure session (O'Daniel and Lee 2012). Given the state of the literature, there is a need for more research on delivery preferences among patient populations. Prior studies have not examined the reasons underlying patients' delivery preferences, whether preferences vary for different types of secondary findings, and how different types of preferences are inter‐related. In addition, preferences could vary if sequencing is conducted in research versus clinical contexts. Research about patient preferences is critical in order to create a feedback strategy for findings generated by genome sequencing that meets patients' needs.

Addressing these questions is most critical for populations for which genome sequencing is already being used. At least in the short term, this technology will likely have the greatest impact for those patients who may carry a highly penetrant disease susceptibility allele (Berg et al. 2011). Young breast cancer patients are therefore an important group for which to develop strategies to return secondary findings from genome sequencing (Pasche and Absher 2011; Ellis et al. 2012). In the United States, breast cancer is the most common malignant tumor in women 15–39 years of age (Johnson et al. 2013); approximately 7% of breast cancers are diagnosed in women <40 years of age (Ries et al. 2006; Brinton et al. 2008). Prior research has shown that women diagnosed with breast cancer at a young age are more likely to carry mutations in *BRCA1* (OMIM: 113705) and *BRCA2* (OMIM: 600185) (Peto et al. 1999; Malone et al. 2000; Bonadona et al. 2005; Musolino et al. 2007; Haffty et al. 2009), and may also carry mutations in other cancer susceptibility genes or novel mutations not previously identified for breast cancer susceptibility (Walsh et al. 2006; Palma et al. 2008; Sakoda et al. 2013). Sequencing is currently being used in the research context to identify additional genes contributing to breast cancer risk.

In addition, genome sequencing findings in the clinical context have increasing potential to affect the care of young breast cancer patients by identifying cancer susceptibility alleles and mutations serving as treatment targets. Among younger women, breast cancer tends to present at a later stage, be more aggressive, and have a poorer prognosis (Anders et al. 2008; Assi et al. 2013). For carriers of known deleterious mutations in cancer susceptibility genes (e.g., *BRCA1/2*), genome sequencing can be beneficial to surveillance and surgical decisions (Ingham et al. 2013; Riedl et al. 2014; Sieh et al. 2014; Heemskerk‐Gerritsen et al. 2015; Trujillano et al. 2015). Sequencing might also be used to identify and choose targeted therapeutic agents for some cancer patients (Ellis et al. 2012; Meiser et al. 2012; Yauch and Settleman 2012; Zardavas et al. 2013). Because of the growing importance of sequencing for this population, which raises a broad array of new ethical considerations, it is particularly critical to examine patient preferences for the delivery of these results so that the needs of patients are met and they can participate in decision making regarding return of results. In a previous study, we examined what secondary findings from genome sequencing that women diagnosed with breast cancer at a young age would like to receive (Kaphingst et al. 2016), but prior research has not yet examined how this population would like the results delivered. To address this important research gap, we investigated in‐depth delivery preferences for secondary findings generated by genome sequencing among 60 women diagnosed with breast cancer at a young age.

## Methods

### Ethical compliance

The university institutional review board prospectively approved this study and all participants provided consent to participate.

### Study participants

We conducted qualitative semistructured in‐person interviews to examine how, when, and from whom women diagnosed with breast cancer at age 40 or younger would like to receive individual genome sequencing results, focusing on secondary findings. We recruited a purposive sample of 60 adults from an existing nationwide cohort of women diagnosed with breast cancer at age 40 or younger, the Young Women's Breast Cancer Program (YWBCP; http://www.siteman.wustl.edu/ywbcp.aspx). The YWBCP cohort is 91% Caucasian; mean age at diagnosis is 35 years and mean time since diagnosis is 7 years at time of enrollment. Because we planned to conduct in‐person interviews, only those YWBCP participants in the St. Louis region were contacted by e‐mail, letter, and e‐newsletter. Interested women contacted the study team to schedule an appointment for an interview.

In order to investigate the preferences of different subgroups of women within the YWBCP cohort, we used a targeted sampling strategy that was stratified by family history of breast cancer, having received prior genetic testing for *BRCA1/2*, and *BRCA1/2* mutation status. This targeted strategy yielded heterogeneity in the sample that allowed us to examine delivery preferences from different perspectives within the patient population (Patton 2015). The four recruitment strata were: women with a strong family history of breast cancer and no identified *BRCA1/2* mutation (*n* = 15); women with low or moderate family history of breast cancer and no identified *BRCA1/2* mutation (*n* = 15); women carrying a known deleterious *BRCA1/2* mutation (*n* = 14); and women who had not received genetic testing (*n* = 16). Family history of breast cancer was scored by an experienced genetic counselor and classified as strong (i.e., one 1st or 2nd‐degree relative diagnosed younger than age 50, two relatives diagnosed at any age, or a male relative diagnosed with breast cancer); moderate (i.e., one 1st or‐2nd degree relative diagnosed at age 50 or older); or low (i.e., no 1st or 2nd ‐degree relatives diagnosed with breast cancer). Study participants were not informed of their family history score.

### Interview procedures

We developed a semistructured interview guide based on existing literature, which was refined based on initial interviews. The interviews began with an introduction to the topic of genome sequencing and a few general questions. Then, open‐ended questions addressed interest in six possible types of secondary results that could be returned from genome sequencing, variants that: (1) related to risk of a preventable or treatable disease; (2) risk of an unpreventable or untreatable disease; (3) affected treatment response; (4) uncertain significance (VUS); (5) carrier status; and (6) no health meaning (i.e., ancestry, physical traits). Each type of possible secondary result was described with examples before the related questions. After discussion of participants' interest in each type of possible result, participants were asked a series of open‐ended questions to explore how they would like individual genome sequencing results delivered, by whom, and when for both research and clinical contexts. These questions about delivery preferences (i.e., how, who, when) were the focus of this analysis. We asked participants to think about their delivery preferences overall and then about differences by type of result.

Two trained master's‐level research staff conducted the interviews, which lasted about 90 min. Interviewers were free to follow the flow of conversation and vary the question order. They were encouraged to probe on responses using follow‐up questions to elicit more detail. All interviews were digitally recorded and transcribed verbatim. Each participant received a $50 gift card for her participation.

### Analysis

The analysis presented here focused on data regarding how, when, and from whom the participants preferred that individual genome sequencing results be delivered. We conducted a directed thematic analysis of the qualitative interview data (Hsieh and Shannon 2005). Initial thematic domains and a preliminary codebook were developed based on prior literature and the interview guide. The codebook was then revised by the research team to add inductively derived codes through an iterative, ongoing process that began after the first interviews were conducted. After the refinement of the codebook was complete, all data were coded with the final codes. Two trained coders independently coded each transcript using NVivo 10, and then met to discuss discrepancies; any remaining discrepancies were resolved by the research team. Analysis was based on consensus codes. After coding, memos summarizing each code were created and used to identify core themes that emerged from the interviews related to participants' preferences for the delivery of genome sequencing results. We first examined themes overall and then whether themes differed across participant subgroups or types of results. We found little difference in themes across participant subgroups or between research and clinical contexts and so present these results overall. We did observe some differences in themes by type of sequencing result, and these differences are described below.

## Results

### Participant characteristics

The current ages of participants ranged between 33 and 64 years. Most (97%) were Caucasian. The majority (73%) had received prior genetic testing for *BRCA1* and *BRCA2*; of these, 14 (32%) carried at least one deleterious mutation. Mean age at diagnosis was 37 years (range 27–40); mean time since diagnosis was 9 years. About 75% had a college degree or higher.

### Preferences for how to deliver results

Most participants (83%) preferred receiving individual genome sequencing results in person, as part of a discussion. This preference for in‐person delivery was often so that the participant could ask questions, thereby getting a better understanding of the information. For example, Participant 50 explained her preference as:

“*Face to face so there would be the opportunity to ask questions. I would not wanna get it in the mail. I would not wanna get it on the phone. I would want a setting… where there's someone there who can tell me what was found and explain it to me; and explain to me what this means; and be available to answer questions that I have*.”

Another participant emphasized the potentially confusing nature of the results and need for explanation as underlying her preference for in‐person delivery: “*Um, I would like to meet with someone simply because I know I would have questions, that's very confusing to me and I would need explanation …. of the significance of the information*.” (Participant 5)

A second common reason for wanting in‐person delivery of results was the desire for help in managing the anxiety that could be generated by the information. For example, Participant 28 said:

“*Definitely in person …… Because I think that it carries a lot of potential anxiety with it, so I think that having that one‐on‐one contact with somebody who really understands it and could kinda talk you through it … I mean that would definitely be my preferred method. …… Not just receiving it in a letter, and feeling really upset and not knowing what it means.”*

About 31% of participants wanted to receive results in a written document: “*I am a reader, so I would love a big fat pack of information that I can read through at my own pace*” (Participant 40). However, many of these participants saw a written document as a supplement to an in‐person discussion. For example, Participant 12 said:

“*I'd like a combination of something in writing, as well as a verbal discussion with somebody to help make it relevant and understand the key things. … But I want to have somebody discuss it all with me because, one, you can always read into things that aren't there, two, you may miss things, and three; it's a matter of helping to manage expectations … as well as to put it in perspective*.”

For some participants, the preference for both in‐person and written delivery of results reflected a desire for a follow‐up process to answer additional questions. Participant 55 described her ideal process as: “*I think it's helpful to have both a human contact and something printed you could take home and read through again. And then … you could call if you had further questions just to explain the, you know, scientific part of it…”*

Some participants had differences in preferences for how the results would be delivered based on the type of result. Participant 14 said: “*I think it depends on the news that's being delivered. I think the ideal, for something complex, where you'd have to really discuss the implications, is a different kind of delivery*.” In general, these participants felt that in‐person discussions were more important for results with high relevance, complexity, or personal impact, whereas written reports were thought to be more suitable for results with less personal relevance or importance to disease risk. For example, in discussing results for variants related to risk of an unpreventable or untreatable disease, Participant 11 said: “*My fears are kinda the Parkinson's and that sort of, or the Alzheimer's. I guess if there was maybe news to deliver or something along those lines, maybe from a counselor, um, backed up by a report*.” Participant 51 described her thought process as:

“ *And if there's something very delicate, for example, if one of my variations shows that, this women, it's amazing she hasn't keeled over from a heart attack already. That, then I'd better get a phone call. If it's not devastating, I guess, I'm okay getting a packet in the mail*. *But if there's information and it's very sensitive and could be very devastating to me or somebody in my family, I want somebody to call me in*.”

### Preferences for who should deliver results

In response to the open‐ended questions about who they would like to deliver individual results from genome sequencing, participants gave a variety of responses, including a researcher (34%), primary care provider (32%), and genetic counselor (31%). For example, Participant 8 responded: “*My doctor. So we would both know and if I had questions*.” In contrast, Participant 53 said: “*Probably a genetic counselor. Because that's their business. They know the most about it*.” For a few participants, the best person to deliver the results depended upon whether the results related to cancer or not. Participant 11 commented “*I guess if it was cancer related from the oncologist…. I guess if it was maybe I'd say anything other than cancer, then maybe by the primary care*.”

However, rather than focusing on a specific role (e.g., primary care provider, genetic counselor), most participants focused on the ideal characteristics of the person who would deliver results. Across participants, the same three characteristics emerged as important: knowledgeable about genome sequencing, ability to explain the results in a way they could understand, and allowing an opportunity to ask questions. For example, Participant 22 commented “*someone who understands what the results mean and can answer my questions*.” Similarly, Participant 29 said “*any of [the possible professionals] as long as they kinda knew what they were talking about, I'd be fine with it. Somebody that knows the information*.” In addition to the information delivery characteristics identified by most participants (i.e., knowledgeable, explains in an understandable manner, answers questions), a few participants highlighted the importance of compassion and empathy in the person delivering the results. For example, Participant 32 said “*someone that has compassion about the situation and not just … a core scientist, but someone that is good with the delivery, good bedside manner*.” Another commented “*I think just somebody who's knowledgeable and caring, who's good at talking to people*” (Participant 28).

In a few cases, participants felt that a team approach might be beneficial. For example, one participant said “*I wouldn't mind if (my doctor) gave it to me and then referred me to somebody that really understood it better than they did, where they could explain it better or answer questions that my doctor might not be able to*” (Participant 24). However, others felt that the person delivering the results should be able to explain the information and answer questions without having to refer the patient on. For example, Participant 52 said:

*“Someone that's knowledgeable about the topic, I mean, obviously like a genetic counselor. You can't get any better than that. Somebody that can answer questions, that's knowledgeable. … A long time ago, I had a test ran. … It was some silly thing that a primary care physician did. And then they gave me the results, and it was just like, ‘If you have any questions then you can go to this, you know, website or call this person.’ I'm like, ‘are you kidding me?’… I mean if you're gonna tell me that I'm gonna have a life‐threatening disease that can't be prevented or treated, I want you be able to explain to me… without having to refer me to someone else*.”

### Preferences for when results should be delivered

Almost all participants thought that sequencing results should be delivered at the time of diagnosis with breast cancer, or if the information was not available then, as soon as possible. For example, Participant 6 commented “*I didn't get [genetic information] at the beginning but I would take it now. As soon as they come out with this, as soon as, it's available*.” Another said “*I guess as soon as possible if they are interested in getting that information… Because it might help in the decision‐making process for screening, prevention, treatment decisions*” (Participant 45). The most common reasons stated for this timing preference were so that women could gain more knowledge and feel better informed and so that they could make the best choices for themselves and their families. Participant 39 explained how sequencing results might impact a range of possible decisions:

“*Because when I was first diagnosed, I just had, I didn't obviously have the genome sequence, but I had genetic counseling and I found out I carried the gene. It made me more susceptible for ovarian cancer. It was kind of like an aha moment, like I need to do something to prevent that. If there's something that they can do to prolong their life, then I think it is beneficial. Just knowing that, it also made my oncologist start doing, with the history of pancreatic cancer, they started doing the pancreatic tumor levels just to keep an eye on them to see what my borderline starting range was, to see, five months ago they went up. What happened? Should we start watching her more closely in that area? I think yeah, in the beginning would be good.”*

Some participants linked a need for information from genome sequencing to their young age at breast cancer diagnosis, stating that younger cancer patients will have different information needs than older patients. For example, Participant 32 said:

“*I think anything you can share would be really, really helpful. I think it's key to understand that, for different points in your life, people need different information and different treatments, because you wanna be super‐aggressive when you're young. You wanna live a long time. You get it when you're 75, you know, it's a different path, very different. Absolutely, as soon as possible.”*

Another participant commented:

“*Well, I think it's important to learn at a young age, because, I think, then they can start planning. I think, um, you know, they can get the options, because some women could maybe have eggs frozen, if they think they're gonna have their ovaries removed. They might choose to have a prophylactic mastectomy, when they could have reconstruction, without waiting until after the fact, and not being, you know, able to have all the options. So, I think it's helpful, when you're younger, because you have more options* (Participant 41).

Some participants did distinguish between different types of results in considering when the information should be returned. For these participants, results relevant to treatment response were seen as most salient at the time of diagnosis: “*I mean when you're diagnosed, anything you can learn to make those treatments more applicable to who you are and what your body makeup is, I think that would be great*.” (Participant 32). Participant 28 described a process for delivery of different types of results over time:

“*Well, I mean, … the part where, about how you respond to medication and treatment would be right up front. That might be the most useful place for that particular piece of the information, because it seems like it make a huge difference in, you know, maybe the course that they would take. Um, and then maybe, um, I mean some of the other pieces could come later. I just know that there's so much information you get right at first, and it's such a total shock that you don't take in a lot, really*.”

In addition to type of result, some participants also believed that the optimal time for results delivery would depend upon factors such as the patient's prognosis and her coping and available social support. Participant 57 commented:

“*There is so much, you're bombarded with so much information at the beginning of your diagnosis, it might be a little bit scary. So, um, I guess the oncologist or whomever is deciding the treatment plan should probably, um, check to see how, uh, mentally, uh, strong and what kind of support system that that young breast cancer, um, patient has. And, um, you know, maybe they'd be able to see if she'd be able to handle all the information at one time, because there's just so much information at the beginning that maybe, uh, maybe they should wait to see when it kind of calms down, so.”*

Because the optimal timing for results delivery was thought to vary for different patients, some participants emphasized that patients should have a voice in planning for results delivery.

“*Or at least give them that opportunity to pursue that at the beginning, even before surgery and things like that. I mean, I guess give them the opportunity, and then it has to be a, you know, a personal decision if they wanna do that, because they need to take into consideration all‐I guess themselves and their families and what it might mean, and that sort of thing* (Participant 11).”

## Conclusions

In this analysis, we examined delivery preferences for genome sequencing results among women diagnosed with breast cancer at a young age, focusing on secondary findings from sequencing. The major findings from the study were that: (1) many of the participants wanted individual sequencing results as soon as possible, even at the time of breast cancer diagnosis; (2) participants wanted an opportunity for an in‐person discussion of results, not simply a report; and (3) they put less emphasis on the type of person delivering results than on the knowledge and communicative skills of that person. While we observed some differences in delivery preferences by type of sequencing result, as described above, the different participant subgroups had quite similar preferences. This suggests that clinical differences between patients might not be the most important factor to consider in designing strategies for return of secondary findings, and that the characteristics of various secondary findings are likely more important.

For the timing of results delivery, we found that participants overwhelmingly thought that the results from genome sequencing should be returned at the time of breast cancer diagnosis, and if the information was not available then, as soon as they were available. This preference was based on the importance of feeling informed, particularly as women make choices about treatment. Some participants also mentioned the importance of young women receiving as much information as possible for making lifestyle choices. Some participants did prioritize information salient to treatment response as most critical to receive at the time of diagnosis, with the timing of return of other types of results as more flexible depending on the woman's personal circumstances. It is important to note that, currently, the results from single gene or panel testing typically take two to three weeks from the time of testing, and the turnaround time for whole genome sequencing is likely to be longer. This may limit the utility of genome sequencing results for immediate treatment planning and is something that would be important to discuss with patients at the time the test is ordered. In addition, at the time of diagnosis, it would likely be important to discuss with patients that results from tumor profiling may be informative for treatment decision making.

This preference for receiving sequencing results as soon as possible, even at the time of breast cancer diagnosis, contrasts with prior literature and many expert recommendations. In a study of participants in a clinical sequencing protocol, the majority strongly preferred an iterative process for return of results (Wright et al. 2014). Other studies have indicated a preference for flexibility in timing of return of results (Tabor et al. 2012; Grove et al. 2014). Some experts have raised concerns that patients may not be emotionally prepared to receive secondary findings at an initial disclosure session (O'Daniel and Lee 2012). Researchers raised similar concerns for rapid genetic counseling and testing for known cancer susceptibility genes, noting that this approach may add to cancer patients' psychological distress or lead to information overload (Ardern‐Jones et al. 2005; Francken et al. 2013; Hall et al. 2014). However, research with breast and ovarian cancer patients to address this issue has shown that although rapid genetic counseling and testing may cause some distress, women generally found the information to be acceptable and beneficial in making cancer decisions (Schlich‐Bakker et al. 2008; Meiser et al. 2012; Francken et al. 2013). This literature has also suggested that some women would not want secondary findings returned until after their treatment decisions were made (Gleeson et al. 2013). Differences in personal utility for various types of secondary findings might also affect timing preferences and this is an important question to examine in future studies.

Research is needed to examine patients' cognitive and affective responses to the return of secondary findings from genome sequencing, and how these responses may differ according to the approach to results delivery. In addition, prior research has suggested that women may consider the way that information is presented to be more important than the timing (Ardern‐Jones et al. 2005). This finding suggests that it will be critical to consider all delivery preferences together in planning strategies for return of results.

In this study, we found that participants had a strong preference for receiving genome sequencing results in person, sometimes supplemented by a written report. We found that this preference was mainly driven by wanting an opportunity to ask questions about the potentially confusing information and help in managing anxiety generated by the results. We did observe some differences in preferences by type of result, with in‐person delivery thought to be most important for complex results with high personal relevance, such as variants that affected risk of unpreventable or untreatable disease. Preference for in‐person delivery is consistent with findings from focus groups conducted with participants in a clinical sequencing protocol (Wright et al. 2014). In focus groups conducted with the general public, face‐to‐face delivery was also most popular for return of results from exome or genome sequencing, although participants wanted options including mail, phone, and Internet delivery (Yu et al. 2013).

However, other studies report preferences for how to deliver genetic results that differ from ours. Participants in a family study of bipolar disorder had a preference for delivery of genome sequencing results by letter or phone (Bui et al. 2014). In another study, most parents said that they would use a confidential website to obtain genetic research results (Fernandez et al. 2014). This implies that the context and potential social acceptability of the disease (i.e., mental illness) may be a factor in these preferences, but this is not well studied. Prior studies have rarely examined the reasons underlying delivery preferences. However, it is possible that differences observed across studies may be due to differences in participants' experiences with genetic testing or receipt of genetic information. Most of the participants in our study had prior experience with cancer genetic testing. Their previous experience may have led them to value assistance with interpreting genomic results and managing emotional reactions. They might also have more knowledge about genetic testing and genetic services than patients without prior experience with genetic testing, which could impact their preferences for delivery of results.

The findings from our study revealed less consensus regarding from whom participants preferred to have genome sequencing results returned, with equal proportions identifying genetic counselors, primary care providers, and researchers. Few identified an oncologist as their preferred person to deliver results. However, there was more consistency on the communication characteristics that this person should have: knowledge, ability to clearly explain results, and allowing questions. These findings are consistent with those of other studies. In focus groups with non‐African American participants recruited from the general public, multiple types of clinicians (e.g., doctors, genetic counselors) were similarly identified (Yu et al. 2014). Our findings are also consistent with prior research in which different patient populations have prioritized having a knowledgeable, experienced clinician return results from genome sequencing (Levenseller et al. 2014). Other studies have shown a preference for a clinician with genetics training who can help interpret the various results and answer questions (Hitch et al. 2014). Considering together the related delivery preferences for how genome sequencing results are returned and by whom, our findings suggest that women diagnosed with breast cancer at a young age preferred in‐person delivery by a genetics expert or another clinician with specialized experience in genetics. Our findings showed that patients also prioritized delivery by a person who is empathetic and can communicate well. Patient perceptions of these attributes are an area of interest for future research.

This preference is concordant with common current practices in return of genome sequencing results. A survey of genetic counselors showed that patients primarily receive results from sequencing in person (Machini et al. 2014). Expert recommendations for in‐person delivery of sequencing results in combination with a written summary (Levenseller et al. 2014) have emphasized the communicative and interpretive skills of a clinician (Townsend et al. 2012). Several professional organizations have recommended the involvement of genetics professionals in the delivery of genetic testing services (Robson et al. 2010; Riley et al. 2012). As the use of genome sequencing expands, however, this approach is likely to put a substantial strain on the system (McBride et al. 2010; Jarvik et al. 2014), both because of the increased number of patients receiving results and because genome sequencing may require multiple sessions or longer disclosure sessions than returning results from a single‐gene test (Levenseller et al. 2014; Lohn et al. 2014; Pal et al. 2014). The trend in other areas of cancer genetic testing has been toward telephone delivery rather than in‐person (O'Daniel and Lee 2012), with some movement toward accessing genetic test results online (Haga et al. 2014). It is likely that alternative approaches to the delivery of genome sequencing results will also develop as the practice becomes more common.

Research is therefore needed to examine approaches to supplement in‐person delivery of genome sequencing results and patient preferences related to these approaches. For example, as suggested by the participants in this study, return of high impact results may be most critical for in‐person delivery by a genetic counselor. However, some types of results may eventually be returned by a primary care provider or interactive, web‐based format. Many physicians today lack specialized genetics training and are thus unprepared to deliver genome sequencing results (Guttmacher et al. 2007, 2010; Plon et al. 2011; Townsend et al. 2012), potentially leading to misinterpretation of findings (Domanska et al. 2009; Jbilou et al. 2014; McLaughlin et al. 2014). However, with training and support from genetics specialists, primary care physicians may be able to serve in this role (McLaughlin et al. 2014; Vassy et al. 2014). Other experts have suggested the use of online tools (Townsend et al. 2013; Lohn et al. 2014). Computer‐based approaches can increase genetic knowledge (Meilleur and Littleton‐Kearney 2009), and may therefore be useful in patient education associated with return of genome sequencing results. Research comparing the outcomes and acceptability of return of genetic test results by different formats is critical but still limited (Haga et al. 2014; Kinney et al. 2014; Schwartz et al. 2014). Patient involvement in the development and testing of approaches for the return of genome sequencing results will help to ensure that the features important to patients (e.g., clear explanations, opportunities to ask questions) are included.

The findings should be interpreted in light of the study's limitations. We examined how women diagnosed with breast cancer at a young age would prefer to have sequencing results delivered, but subsequent studies are required to test cognitive, affective, and behavioral responses to different strategies for returning these results. Our participant sample was largely Caucasian and preferences may differ by race and ethnicity (Meiser et al. 2012; Yu et al. 2013). These participants had a high educational attainment on average and information processing and patient preferences may differ for population subgroups with lower socioeconomic status and lower educational attainment or health literacy. The findings suggest that preferences were driven in part by the young age of these women at the time of diagnosis; young age may involve different life factors and stressors (e.g., young children, education, working) than those facing women diagnosed at an older age (Greaney et al. 2015). In addition, because young women are more likely to get aggressive cancer treatments than older women, their experiences of these treatments may differ, potentially influencing these views on what information would be valuable at what times and how capable they would have been of handling that information. Therefore, it will be important to explore preferences for return of genome sequencing results among other patient populations as well. We saw few differences in delivery preferences between research and clinical contexts in this study, but preferences may vary in practice between different types of research studies and clinical care.

Despite these limitations, this study adds to our understanding of preferences for return of results from genome sequencing, including secondary findings, among women diagnosed with breast cancer at a young age. Overall, women preferred receipt of the information as soon as possible with a strategy of in‐person delivery of results by a clinician with specialized genetic knowledge. However, another theme that emerged through these findings is the importance of making this a process tailored to the woman's individual circumstances and one that she has a voice in determining. A critical goal for future research will be to develop processes to help patients make decisions about how they would like various results from genome sequencing returned and assist patients in distinguishing between results related to the primary indication for testing and secondary findings that are not related to this indication. One possible approach would be shared decision making, in which providers and patients decide together. An alternative approach would be that providers would participate in education and discussion with patients about their options but that patients would have autonomy to make the decision about return of results. An important direction for future research would be to examine different decision‐making approaches for this issue. A transdisciplinary approach in which communication and patient education experts partner with patients, genetic counselors, clinical geneticists, and other health care providers to develop and test innovative approaches to decision making and return of results will allow us to meet patients' preferences and needs.

## Conflict of Interest

The authors have no conflicts of interest to declare.
